# The Influence of Oral Irritation in the Production of Disease of the Upper Air-Tract

**Published:** 1889-08

**Authors:** Frank Hamilton Potter

**Affiliations:** Buffalo, N. Y., Lecturer on Diseases of the Nose and Throat in the Medical Department of Niagara University; 273 Franklin Street


### *THE INFLUENCE OF ORAL IRRITATION IN THE PRODUCTION OF DISEASE OF THE UPPER AIR-TRACT.*[2]

By FRANK HAMILTON POTTER, M. D., Buffalo, N. Y.,

Lecturer on Diseases of the Nose and Throat in the Medical Department of Niagara University.

THE invitation of your committee to read a paper before the Eighth District Dental Society was very much appreciated, and it was accepted because of the belief that both your specialty and mine could derive benefit from a discussion of some of their interdependent relations. You have so far perfected the knowledge of the diseases of the teeth and their treatment as to have merited and obtained recognition throughout the world, while those of us who are devoting ourselves to the study of the diseases of the upper air-tract—including, as it does, the nose, throat, and, to a large extent, the ears—are fast arriving at a position of positive knowledge concerning many of the maladies seen in this region.

The organs whose diseased conditions come under our notice are situated close together, and it would not be surprising to find that where one was diseased, the others would, to some extent, be affected

---

1. See an article on the Germs of Yellow Fever lately published in *Medical Times and Register* of Philadelphia.

2. Read by invitation before the annual meeting of the Eighth District Dental Society of the State of New York, April 16, 1889.

also. Moreover, the mucous membrane lining the mouth, nose, throat, and the various sinuses connected with them, is continuous, thus inviting the spread of pathological processes. And yet, of far more importance is the intimate nervous supply that binds these diversified organs so closely together. The same nerve, practically, supplies common sensation to all the organs we are considering. This wonderful fifth nerve sends sensory branches to the eye-ball, lachrymal gland, conjunctiva, nasal mucous membrane, all the muscles and integument about the eye-ball, orbit, os frontalis, nose, mouth, cheek, lips, temple, superior portion of the pharynx, tongue, gums, and teeth. Acting under a well-known law of nervous force, a disease in any part supplied by one of these branches may be reflected to a part supplied by another branch, and there produce not only irritation but, occasionally, disease itself. And it is now also well understood that sensory impulses originating in one set of nerves may flow through the sensory tracts in the medulla and pons, and be reflected to parts supplied by an entirely different set of nerves. Thus distant sources of irritation often play an important part in the etiology of disease. What nervous force is, we do not know, but many of its phenomena we can understand. We know, for instance, that nasal disease frequently produces cough and other symptoms located by the patient in the larynx. Sometimes they will insist, on account of this cough, that they have lung trouble, but the symptoms will disappear on the correction of the nasal difficulty. There is also a cough produced by ear disease, and is known as ear-cough. And it is familiar to all dentists that the various neuralgias of the head and face may be aggravated or even produced by diseased teeth. In certain conditions of the system, the nerves become exceedingly impressionable; and, as beautifully stated by Sexton, "When nerve-tension has been long disturbed in this way, reflex phenomena are easily excited; continuous aural, nasal, or dental irritation, even if imperceptible, may affect one part or another, until nutritive (trophic) changes are brought about."

The reflexes having their origin in diseases of the nose, and throat, and ears, have been studied, to some extent, and a number of very interesting cases are on record illustrating them. In some instances, no doubt, the reports of these cases may be questioned, the enthusiasm of the observers having carried them too far. But enough remains to demonstrate the importance of a careful study of the subject. Dr. John N. Mackenzie, of Baltimore, in a paper on the "Pathological Nasal Reflex,"[1] says:

1. Transactions American Laryngological Association, 1887.

"Various neuralgic conditions of the branches of the fifth and other nerves—cough, asthma, vertigo, nightmare, 'hay-fever,' various spasmodic affections, genera. convulsions, diseased states of the nose, eye, ear, larynx, and bronchiai ubes, symptoms referable to irritation of the gastro-intestinal, utero-ovarian, and genito-urinary tracts; even chorea, epilepsy, melancholia, retarded sexual development, and exophthalmic goitre—have been mitigated, or known to disappear, with the cure of the nasal affection."

While so much has been done in this and other directions, the teeth, as the starting-point of reflex irritation in the production of catarrh, have not received the attention they deserve. The writer's attention was first called to this subject by cases occurring in his practice, and while his studies are not yet, in many instances, complete, sufficient has been observed to warrant the statement that in cases of "catarrh" the health of the teeth should be carefully inquired into. Of course, there may be, and generally are, other causative factors at work, but this, at least, should not be neglected.

Let us first inquire into the sources of oral irritation, and then consider the reflex phenomena resulting therefrom.

The subject of oral irritation has been very carefully studied by Dr. Samuel Sexton, of New York, to whose work I am indebted for much that is of great value. His investigations had to do with diseases as seen in the ears, but his discussion of the question is broad, and can be utilized with respect to other regions of the head subject to catarrhal inflammation. The eruption of the teeth, as is well known, frequently causes severe irritation, both of a local and of a reflex nature. Teething children often present repeated attacks of a subacute coryza, the discharge from the nasal passages being of a watery nature. If possible to make an examination, the mucous membrane will be found swollen and red. This may not be sufficient to obstruct nasal respiration, and indeed, in my experience, it usually has not done so, and differs, in this respect, from an ordinary severe cold. Congestion may also occur in the ears, and give rise to earache, and this may go on to a severe inflammation attended with more or less danger to hearing.

Second dentition may produce like results, the rhinorrhea and otorrhea requiring constant attention. In this case, search should be made for small fragments of the milk-teeth that may adhere to the cavities, and if found, they should, of course, be removed. The eruption of the wisdom teeth often cause severe disturbances. There may be inflammation of the pharynx and tonsils, and also of the gums. The jaw-bones may be involved, and even necrosis occur, from the severity of the inflammation. The nose, too, occasionally shows the influence of this irritation. There may be developed an obstinate rhinitis, or, if there is any preëxisting catarrh, this may be greatly aggravated. Affections of the autrum of Higlimore are not uncommon, and Sexton says that some of the most protracted and intractable cases of acute purulent inflammation of the middle ear he has ever seen, have been associated with the cutting of a wisdom tooth.

The eruption of the teeth is a physiological process, and, therefore, the resulting irritations cannot be said to depend upon diseased conditions. We have now to inquire what diseases may give rise to these irritations. In cases of catarrh, we should ascertain the condition of the teeth (*a*) as to the indications of inherited disease, as scrofula, syphilis, and the like; (*b*) as to the collections of tartar upon them; (*c*) as to any sharp processes that may produce fissures or ulcers on the tongue or in the mouth; (*d*) as to their soundness, whether they are loose or exhibit any evidence of necrosis; (*e*) as to whether there are any concealed fangs left on extraction; (*f*) for completeness or deficiency in number, in cases of dysphagia, to be possibly accounted for by imperfect mastication, "bolting" of the bolus, and consequent fatigue and paresis of the pharyngeal constrictor; and (*g*) for the crowding or any irregularity in their formation.

These are some of the diseases affecting the teeth that require correction; other irritations arise or are dependent upon incomplete or illy-directed efforts to correct them. I refer to the poor adaptation of dental plates or fillings. This subject is of no less importance than the diseased states themselves. Fillings that are so badly adapted as to irritate the gums or lips, may produce harmful results. There have been cases also where the fillings were placed in cavities before the necrosed parts were thoroughly removed. In this way, they shut up foreign particles that give rise to most distressing reflex disturbances. Plates, also, that are not well moulded to the parts are serious evils. If placed upon carious fangs, or over-inflamed gums, they not only increase the irritation by contact, but prevent the escape of the foul secretions, and thus expose the patient to septic poisoning. The material used in the construction of the plates may give rise to reflex troubles. It is stated that the vulcanite plates are dangerous, especially when colored by the sulphide of mercury.

Many other sources of irritation will, no doubt, occur to you. These are mentioned to illustrate, in a general way, the proposition that, as the sources of irritation are numerous, the reflex phenomena resulting therefrom are also of great number. What are some of these reflex disturbances? In the first place, there is pain—the various neuralgias of the face and head—pain also in the throat, ears, and even in the chest. Cough is not infrequent. Acute inflammation may result, or a chronic inflammation be aggravated. This may be seen in the larynx, pharynx, and tonsils, in the nose and the ears. In the nose, there may be a simple swelling of the mucous membrane, persistent in character, and giving the unpleasant sensation of "stuffiness;" or a muco-purulent inflammation may be observed. The autra of Higlimore may be involved, and give rise to distressing symptoms. In the ears, we may have trouble ranging from pain to an acute purulent otitis. In the catarrhally disposed, or where there is any existing disease of these parts, reflex disturbances are more easily manifested. Chronic disease is aggravated, and not infrequently acute attacks are superimposed. In this latter case, of course, the symptoms are more intense, and relief more difficult.

A description of these diseases would be out of place in this paper. It is sufficient to simply call attention to the causative relation the teeth bear to them.

Thus far we have considered that an oral irritation may develop or aggravate a catarrhal inflammation of the upper air-tract; let us now inquire if the contrary may not be true also. Will not disease of the nose, throat, and mouth hasten the development of disease in the teeth? To this question you would be more competent, perhaps, to give a satisfactory answer. Considered theoretically, it seems not only possible but probable. And, indeed, my observations lead me believe that it is true. Certain it is that the patient with catarrh, if questioned closely, will often tell you that the teeth seem to decay very rapidly. Whether the disease of the teeth or the disease of the other organs appeared primarily or not, or whether they are both dependent on some general cause, may be difficult to determine in a given case, but it often can be determined in a satisfactory manner. I believe that if more attention was given to disease of the upper air-passages, including with them the mouth and ears, there would be less disease found in the teeth.

In considering this subject, therefore, we see that a duty rests upon the dentists as well as with us. When patients come to us with any disease in the air-passages, we should in our examination include the condition of the teeth. If they are found to be diseased or malformed, we should advise them to have such disease or malformation corrected, at the same time explaining our reasons for such advice. On the other hand, when you observe patients to suffer from any of the many troubles we have referred to, they should be told the importance of having such disease properly treated. Not only should this be so, on both sides, from the influence that these diseases may have the one upon the other, but also the influence they may have upon the general health. This latter subject is referred to incidentally, as it is without the scope of this paper, but should always be borne in mind.

In closing, I desire you to consider this paper as suggestive. It does not aim at an exhaustive presentation of the subject. The literature referring to it is too slight, and my own observations not yet sufficiently formulated, to accomplish that. This may be called a preliminary note, suggesting a standpoint from which to pursue further studies. The causes of disease are complex and subtle, and while we do not yet know them all, let us follow any light that may help us to a better understanding of our work.

The conclusions of this paper may be presented as follows:

I. Oral irritation is a factor in the causation of disease of the upper air-tract.

II. This irritation results from any disease or malformation of the teeth, and also from an incomplete and improper adaptation of fillings and plates.

III. Disease of the upper air-tract may be a causative factor in the production of the diseases of the teeth.

IV. The recognition of the reflex influence of the diseases of these organs is an important step in their diagnosis and treatment.

273 FRANKLIN STREET.

